# Mitigation of Volume Changes of Alkali-Activated Materials by Using Limestone Filler

**DOI:** 10.3390/ma18132963

**Published:** 2025-06-23

**Authors:** Maïté Lacante, Brice Delsaute, Stéphanie Staquet

**Affiliations:** 1 BATir Department (LGC), Université libre de Bruxelles (ULB), Avenue Franklin Roosevelt 50, 1050 Brussels, Belgium; maite.lacante@ulb.be (M.L.); brice.delsaute@ulb.be (B.D.); 2 CRIC-OCCN, Avenue A. Buyl 87, 1050 Brussels, Belgium

**Keywords:** blast-furnace slag, limestone filler, compressive strength, coefficient of thermal expansion, autogenous strain

## Abstract

As autogenous and thermal strains are significantly high in alkali-activated pastes, it becomes necessary to investigate ways to reduce these. This research studies how the volume changes of pastes made from slag activated by alkalis can be mitigated by substituting part of the slag with limestone filler and how this impacts the properties of the material, including autogenous strains, thermal strains, heat flow, compressive strength, and workability. The first part investigates how the different substitution rates impact the compressive strength and workability. The substitution rates of 15% and 30% emerged as the most optimal with a maximal reduction in the compressive strength of 23%. Five compositions were consequently investigated in the second part of the study. Isothermal calorimetry revealed that the limestone filler was probably not entirely inert and showed the effect of dilution, which is linked to the increase in the solution-to-binder ratio when the substitution rate increases. The autogenous shrinkage decreased when substituting 15% of the slag, while higher autogenous shrinkage was obtained when 30% was substituted. In addition, its rate of development was reduced. Finally, the coefficient of thermal expansion was generally slightly reduced and delayed when slag was substituted.

## 1. Introduction

Due to its durability, fire resistance and versatility, Portland cement concrete is the most widely used material in the construction sector. It consists of aggregates, sand, Portland cement (PC), and water, with additives sometimes included for enhanced performance. Unfortunatly, Portland cement’s production significantly contributes to global CO_2_ emissions (5–8%) [1,2,3]. A key strategy to reduce PC’s environmental impact is the use of alkali-activated materials (AAMs) [4,5]. These alternatives to PC paste seem to be a good solution considering their ecofriendliness, excellent thermal resistance, as well as their durability and resistance to fire. However, one of the disadvantages is the significant change in volume at early age, which inevitably creates internal tensile stress, possibly leading to the microcracking of the paste. Therefore, it creates a brittle concrete material, highlighting the need to thoroughly investigate the changes in volume at early age.

Previous research has demonstrated that alkali-activated slag pastes exhibit significant volume changes, including autogenous shrinkage and thermal strains due to their high coefficient of thermal expansion (CTE), highlighting the necessity of mitigating these effects [6]. Studies on curing temperature have shown that while increasing the curing temperature reduces autogenous shrinkage, it also leads to an increased CTE and, consequently, higher thermal strains [7]. Therefore, this study focuses on the partial use of alternative materials in order to reduce these volume changes. For this purpose, limestone filler as well as metakaolin may be an option [8,9,10,11], as well as enhancing carbonation resistance and chloride binding capacity due to the resulting phase modification [12]. Limestone filler, in particular, is abundant in the south of Belgium (Wallonia), making it a cost-effective option as well as a local resource.

Limestone filler exists as a dry powder. Its grain fineness varies by type, influencing flow behavior. Other factors, such as grain morphology and affinity with the liquid phase, also affect the flowability properties. The affinity of filler with liquids depends on the grain surface characteristics, which impact the material’s rheology [13]. Mortars containing PC show reduced flowability when the limestone filler substitution increases, likely due to the altered packing of fine particles. The flowability decreases at a 15% substitution rate but increases when more cement is replaced. In ternary composites, higher limestone filler substitution enhances flowability [14,15].

Limestone filler, often considered inert, can enhance the rheology of alkali-activated materials through a filler effect and can thus be considered as physically active [16]. When mixed with a sodium silicate-activated slag–fly ash paste at substitution rates of 5–20%, limestone filler reduces both yield stress and plastic viscosity [14,17]. Workability as well as the compressive strength is influenced not only by the substitution rate but also by the solution-to-binder ratio. The workability increases as the (alkali-)solution-to-limestone-filler ratio increases while the compressive strength decreases [18].

Studies show that limestone filler has been widely accepted in many standards to replace PC, with a maximum limestone filler content acceptable of 5 to 15%. However, only a few studies have investigated substitution with limestone filler in AAM. Rashad et al. [19] found that a 15% substitution rate in AAM resulted in an increase of 11% of the compressive strength while higher substitution rates decreased the compressive strength. Substitution rates of up to 50% have been investigated, showing an increase in the autogenous shrinkage for up to 15% mass substitution [20] and 30% volume substitution [21] while a reduction was found for a 50% volume substitution [21]. This increase is linked to the increase in the gel pore content which decreases the mesopore density. This results in a higher driving force of autogenous shrinkage [20]. Conversely, the decrease in autogenous shrinkage can be related to the dilution effect and reduction in reaction kinetics [21].

Replacing the binder by limestone filler can create physical changes, such as modification of the particle size distribution, heterogeneous nucleation, and dilution [22,23]. The limestone filler has an increasing effect on the compressive strength of pastes because its addition to the mix accelerates the reaction while reducing the porosity. In fact, limestone filler has finer grains compared to slag and it allows the voids to be filled. The addition of limestone filler provides an additional surface area for the nucleation of the products from the reaction. Moreover, the energy barrier is reduced, which allows the reaction products to precipitate faster [22,24,25]. In cement mortar, limestone filler might chemically contribute to the formation of mono-carboaluminate. This reaction product increases the strength of the material [14,26]. However, some contradictions have been observed in studies which showed that ternary PC pastes composed of slag and limestone filler showed a decrease in their compressive strength at early age when the limestone filler substitution rate increased up to 22% [27,28]. The dilution effect occurs when a used binder is partially substituted, resulting in an increase in the solution-to-slag ratio. In the case of cement compositions, clinker substitution by limestone filler decreases the compressive strength of the paste partially because of the increased water-to-cement ratio [23]. With a small amount of limestone substitution ranging from 5% to 15%, the compressive strength of PC concrete already increases at early age. It was observed that limestone filler could help at early age while slag increases the long-term strength [14,27].

**Figure 1:**
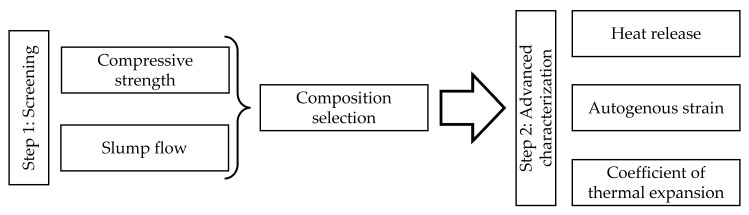
Steps of the two-part study.

The present study explores the use of limestone filler as a partial substitution of slag in order to attempt to mitigate the volume changes. This research was conducted in two parts (see Figure 1). The first part consists of a preliminary campaign followed by an in-depth testing campaign. The preliminary phase serves as a selection process in which a range of compositions are proposed and evaluated in terms of workability and compressive strength performance. These compositions were chosen based on a literature review and examine the replacement of blast-furnace slag with limestone filler (LF) at various percentages. Ultimately, five compositions were selected for further detailed investigation in the second part where the reaction kinetics, the autogenous strain, and the thermal strains are studied.

## 2. Materials and Methods

### 2.1. Materials

A previous study investigated pastes made from blast-furnace slag (BFS) which were activated with sodium hydroxide, referred to in this study as the reference compositions [29].

Regarding the activating solution, the primary focus is on sodium hydroxide. In addition, in order to assess the activator’s influence on these properties, sodium silicate (10 M) is also used.

Each material’s chemical composition is reported in Table 1. The Blaine fineness of the limestone filler (LF) is 4580 cm^2^/g while that of the slag is 4690 cm^2^/g. The specific gravity of limestone filler is 2.69 g/cm^3^ and that of the blast furnace slag is 2.87 g/cm^3^.

### 2.2. Compositions

#### 2.2.1. Reference Composition

The reference compositions are presented in Table 2 and were previously studied in Lacante et al. [29]. In the case of slag activated by sodium hydroxide, the primary internal parameters (solution-to-binder and concentration of the alkaline solution) impact the development of the autogenous strain in alkali-activated materials [30], similarly to the impact of the water-to-cement ratio on these properties in cementitious materials [31]. The solution-to-binder (S/B) ratio is equal to 0.5 and 0.8 because compositions with lower S/B have poor workability [30], while a higher S/B increases the bleeding risks. The NaOH molar concentration is 2 and 8 molar. These are realistic molarities because a lower concentration brings a lower reaction rate, which can be insufficient for strength development. A higher concentration increases the reaction and the temperature. Both the temperature of the solution during the preparation and of the reaction of the material increase significantly as the concentration increases. The setting times are also related to the concentration that is used [7]. Finally, after an optimum alkali content (3.57% = paste with S/B of 0.5 and solution concentration of 2.52 M) is reached, increasing the alkali content results in an ultimate heat increase [6].

In the term solution-to-binder, the solution is equal to the sum of the weight of the alkaline solution and of the weight of the water (if it is used). The binder is the sum of slag and the substitution material (=limestone filler) when it is used.

#### 2.2.2. Limestone Filler Substitution

The limestone filler compositions can be divided into five groups, which are represented in Table 3. Category A corresponds to the reference composition P-S05M2, Category B to the reference composition P-S05M8, Category C corresponds to the reference composition P-S08M2, Category D corresponds to the reference composition P-S08M8, and finally, Category E corresponds to the reference compositions with sodium silicate.

Based on the literature study presented in the introduction, the recurring substitution ratio in cement was between 5 and 15%. However, higher substitution rates up to 50% have been investigated as well. Therefore, it was decided to investigate a broad range of substitution ranges going from 10 to 50%. The substitution rate was progressively increased by steps of 10% up to 50% for each of the compositions. In addition, two extra compositions with a 15% substitution rate were added for categories A (reference composition P-S05M2) and B (reference composition P-S05M8). For the compositions with sodium silicate (category E), substitution rates of 30% and 50% were tested for the solution-to-binder ratios of 0.5 and 0.8, respectively. In addition, two types of sodium activators were used in order to prepare the alkaline solutions: NaOH and Na_2_SiO_3_. For the analysis of the results, the binder is equal to the sum of the weight of the slag and of the weight of the limestone filler.

### 2.3. Methods and Materials

#### 2.3.1. Preparation

The preparation of the paste was based on the European Standard EN 196-1:2016 [32].

#### 2.3.2. Slump Flow

The pastes’ workability was evaluated in accordance with the ASTM C230 standard [33]. Immediately after mixing, the paste was poured in the slump flow cone. The opening at the bottom was 100 mm and at the top it was 70 mm. The height of the cone was 50 mm. Subsequently, the cone was lifted. The diameter of the obtained disc of material was measured two times. The measurement was performed with a precision of ±5 mm. There was an angle of 90° between the two measurements. The reported results represent the mean value of these two measurements and the error bars shown represent the minimum and maximum measurements taken. The test was carried out in the lab at (20 ± 2) °C on a metal plate which had been humidified with a wet cloth beforehand. Workability assessment served as a quality control measure for the slag. This helps to assess the degradation level of the material as well as to maintain similar mixing conditions during the whole testing campaign.

#### 2.3.3. Compressive Strength

The pastes’ compressive strength was determined using a cubic sample with sides of 50 mm. The material was cast in appropriately sized molds and vibrated to eliminate air bubbles. The molds were then sealed with a plastic sheet and placed in a climatic chamber to be cured at 20 °C. Following previous studies [6,29], two specimens per composition were tested at each age of 1, 2 and 7 days. Testing was carried out on a 600 kN hydraulic Galdabini press with a 1 kN sensitivity. The procedure followed the ASTM C109 standard [34]. The loading rate was between 900 and 1800 N/s (achieved before the second half of the expected failure load was obtained and without further adjustments). The presented results exhibited a variation below 7.6%, as prescribed by the ASTM C109 requirements [34].

#### 2.3.4. Apparent Density

The apparent density was calculated based on the cubes used for determining the compressive strength of the paste (see next section). Their weight as well as their dimensions were taken, with a maximum variation of 4.35% between samples of the same age, while the average error was 1.07%. Since no clear trend was observed over time, the measured densities presented in the corresponding section are the mean of the measurements (with maximum time variation below 8.73%, while the mean error was 4.05%).

#### 2.3.5. Isothermal Calorimetry

The reaction of the alkali-activated slag and limestone filler pastes was followed with an isothermal calorimeter (TAM Air). The device is based on the European standard EN 196-11:2018 [35]. This calorimeter features eight channels that operate at the same time and at the same temperature. Each one of these channels can accommodate two ampoules. One ampoule is the sample to be tested (containing about 7.5 g of the material under investigation) and the other ampoule contains an inert reference (sand). Each position is equipped with an independent heat flow sensor, minimizing noise and enhancing measurement stability [36].

The heat flow of such materials exhibits two peaks. The initial peak, associated with the slag dissolution is often not monitored due to its rapid occurrence and the outside mixing process. This peak is related to the reactants dissolution, particularly the breakdown of bonds in the slag, the particles’ wetting, as well as the formation and interaction of units of silicate with Ca^2+^ and Na^+^ ions [37,38,39]. The measured peak, which is actually the second reaction peak, is related to the reaction products formation, such as calcium aluminosilicate hydrates [37]. The interval between these peaks is called the “induction period” [40].

Mixing was performed in accordance with the European standard EN 196-1:2016 [32]. After which, the material was poured in the ampoules which were sealed and placed within the calorimeter within the first 10 min after mixing began. Each composition was tested using two ampoules. Regular isothermal calorimetry ensured blast-furnace slag quality control, preventing degradation and maintaining consistency throughout the testing campaign.

#### 2.3.6. Autogenous Strains and Coefficient of Thermal Expansion

The corrugated tubes procedure outlined in ASTM C1698–09 [41] were revisited at the Université Libre de Bruxelles [42] in order to assess the autogenous strains as well as the thermal strains at the same time.

Each test involved imposed cycles of temperature, which were ±3 °C around the 20° curing temperature. The strain measured at time *t* ($\epsilon_{tot}\left(t\right)$ [µm/m]) is the sum of the autogenous strain ($\epsilon_{auto}\left(t\right)$ [µm/m]) and of the thermal strain ($\epsilon_{thermal}\left(t\right)$ [µm/m]) at that same time. The thermal strain is related to the coefficient of thermal expansion (α(t) [µm/m/°C]) and to the temperature variation in the specimen (ΔT(t) [°C]). This is represented by the following Equation [6,43]:

(1) $$\epsilon_{tot}\left(t\right)=\epsilon_{auto}\left(t\right)+\epsilon_{thermal}\left(t\right)=\epsilon_{auto}\left(t\right)+\alpha \left(t\right)\cdot \Delta T\left(t\right)$$

At very early ages, decoupling these strains was necessary. However, after a certain period, the autogenous strain stabilized over short intervals of time, allowing for CTE calculations within those periods [43]. Because of how the procedure was established, the computations of both strains were performed about every two hours.

If the reader would like more explanation or details about the setup or the computation processes, they are invited to consult Delsaute and Staquet [42] or Lacante et al. [6]. Each composition was tested using at least two samples, accompanied by one dummy sample for temperature monitoring.

## 3. Results and Discussion

### 3.1. Preliminary Campaign

**Figure 2:**
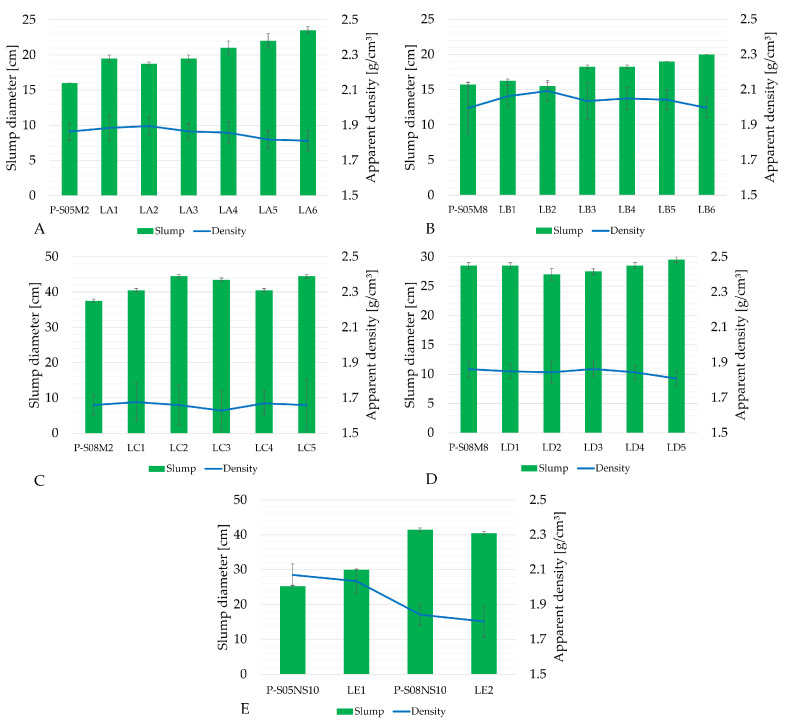
Results of the apparent density and the slump of alkali-activated slag with LF substitution: (**A**) P-S05M2 as reference (category A), (**B**) P-S05M8 as reference (category B), (**C**) P-S08M2 as reference (category C), (**D**) P-S08M8 as reference (category D), (**E**) sodium silicate reference compositions (category E).

**Figure 3:**
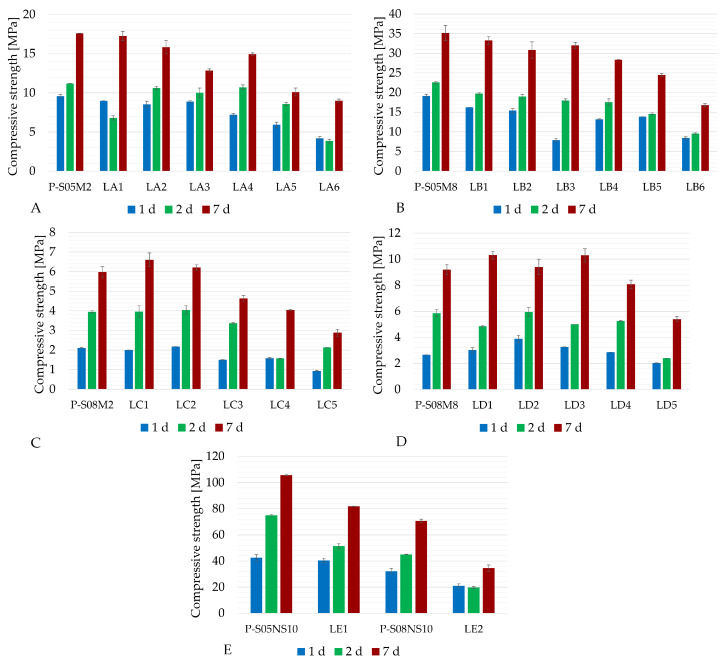
Compressive strength results of alkali-activated slag with LF substitution: (**A**) P-S05M2 as reference (category A), (**B**) P-S05M8 as reference (category B), (**C**) P-S08M2 as reference (category C), (**D**) P-S08M8 as reference (category D), (**E**) sodium silicate reference compositions (category E).

Figure 2 shows the results of the apparent density and the slump measurements for all compositions listed in Table 3, along with a comparison to the reference compositions in Table 2. Figure 3 shows the evolution of their compressive strength over time. At 7 days of age, the percentage reduction in compressive strength compared to the reference pastes was computed depending on the limestone filler substitution rate (see Table 4).

In addition, also at 7 days of age, the percentage by which the compressive strength was boosted due to the addition of limestone filler was computed with Equation (2), where x = substitution rate [-], $f_{c,ref}$ = compressive strength [MPa] at 7 days of the reference composition, and $f_{c,x}$ = 7 days compressive strength [MPa] of the investigated composition with (x·100) % limestone filler substitution. The results can be found in Table 5.

(2) $$\frac{f_{c,x}-\left(1-x\right)\cdot f_{c,ref}}{\left(1-x\right)\cdot f_{c,ref}}$$

A general trend was observed when increasing the quantity of limestone filler in the pastes—substituting slag by limestone filler reduced the compressive strength. In addition, a higher substitution rate increased the slump flow, indicating better workability. The influence of the S/B ratio can be observed by comparing two categories with the same solution concentration: category A and category C both used a 2 M solution (see Figure 3A,C), while category B and category D used an 8M solution (see Figure 3B,D). When the molarity is constant, pastes with an S/B ratio of 0.8 exhibited lower compressive strengths compared to those with an S/B equal to 0.5. However, when 20% and 30% of the slag was replaced with limestone filler for the 2 M and 8 M compositions, pastes with an S/B ratio of 0.8 outperformed the reference paste in terms of compressive strength. The S/B ratio also affected the workability: a higher S/B ratio resulted in increased slump flow. The last observation concerns the influence of the alkaline solution concentration. This effect can be analyzed by comparing categories with the same S/B ratio, such as category A and category B (S/B = 0.5, see Figure 3A,B) or category C and category D (S/B = 0.8, see Figure 3C,D). It can be observed that the higher molarity resulted in a lower slump flow thus reducing the workability, while the compressive strength was increased. Finally, the density of the material was not significantly affected by the substitution rate, with the maximum observed difference being less than 5% of the reference paste.

Concerning the compressive strength, the limestone filler substitution resulted in a dilution effect. For this reason, none of the compressive strengths of the 0.5 S/B compositions exceeded the compressive strength of the reference paste [15,27,28,44]. However, Aqel and Panesar observed an increase in compressive strength when working on cement mortar [24]. The limestone filler addition results in chemical and physical effects. On one side, the finer particles fill the voids present between the larger particles. This can reduce the porosity as well as increase the strength of the material in consequence. Chemically, limestone filler might contribute to the formation of reaction products (similarly to mono-carboaluminate in cement mortar), which can increase the strength of the material, possibly explaining the improved performance when the S/B is increased [14]. This is related to increased reaction and extended chain length of the produced gels as a consequence of the limestone filler addition [12]. On the other hand, pastes with higher contents of limestone filler exhibit lower compressive strengths because of the lower reactivity of calcite present in limestone filler [22]. In general, increasing the substitution rate tends to decrease the compressive strength.

At the same time, the workability improves as the limestone filler substitution is increased [14,15]. Limestone filler has a hydrophilic nature, leading to a more fluid paste [13]. The S/B also significantly affects the workability: a higher S/B ratio results in a greater slump flow and, consequently improved workability. The use of a high-molarity alkaline solution enhances the compressive strength and accelerates the setting time [45]. This is due to the increased concentration of hydroxide ions, which speeds up aluminosilicate gel formation. The only notable difference when using Na_2_SiO_3_ as an activator, compared to NaOH, is the considerably higher compressive strength. However, the overall trends regarding limestone substitution remain the same.

Compared to the reference slags, a higher limestone filler substitution leads to a lower compressive strength but an improved workability. Moreover, an S/B of 0.5 provides better overall performances than a higher S/B. Additionally, increasing the concentration of the activator increases the compressive strength. To investigate the volume changes of alkali-activated paste, five compositions were selected, considering both the substitution amounts and the compressive strength and workability results to achieve a satisfactory compromise. To ensure a relevant comparison, the S/B ratio was kept constant at S/B = 0.5, focusing on categories A and B, which typically exhibit the highest shrinkage [29]. The limestone filler substitutions were set at 15 % and 30 %, representing a reasonable trade-off between compressive strength reduction (of maximum 19.3 %, resulting in 28.4 MPa at 7 days) and enhanced workability while maintaining a noticeable substitution difference. On the other side, the increase in limestone substitution increased the compressive strength of several compositions with an S/B equal to 0.8. However, their compressive strength (maximum 10.3 MPa at 7 days) still remained lower than that of the selected 0.5 S/B compositions (minimum 15.0 MPa at 7 days). In addition, these showed lower autogenous shrinkage [29]. Therefore, these were not considered in the present study. Lastly, a composition with sodium silicate as the only activator was selected: LE1, as it maintained the same S/B ratio of 0.5 and achieved the highest compressive strength of the compositions with limestone filler (82 MPa at 7 days) despite a 23% compressive strength decrease.

### 3.2. In Depth Investigation

#### 3.2.1. Reaction Kinetics with Isothermal Calorimetry

The first step of the in-depth analysis of the limestone filler compositions selected in Section 3.1 is the investigation of reaction kinetics. For this, the heat flow and cumulative heat were monitored by means of isothermal calorimetry which follows the (exothermic) reaction of the material.

**Figure 4:**
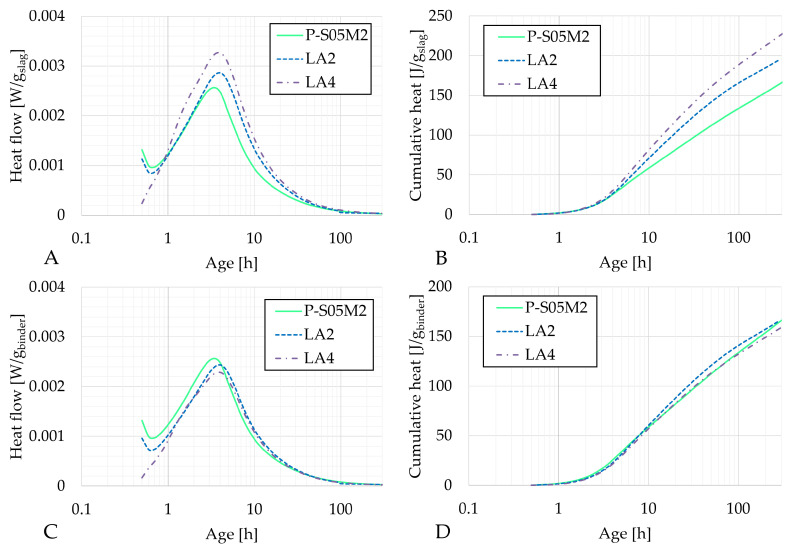
Results of the reaction kinetics followed through isothermal calorimetry for P-S05M2, LA2 and LA4: (**A**) Heat flow and (**B**) cumulative heat presented per gram of slag; (**C**) Heat flow and (**D**) cumulative heat presented per gram of binder (=limestone filler+slag).

**Figure 5:**
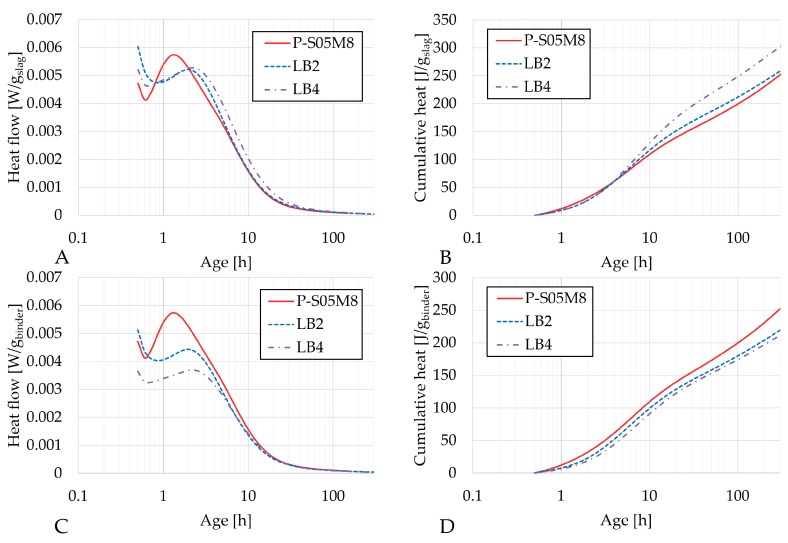
Results of the reaction kinetics followed through isothermal calorimetry for P-S05M8, LB2 and LB4: (**A**) Heat flow and (**B**) cumulative heat presented per gram of slag; (**C**) Heat flow and (**D**) cumulative heat presented per gram of binder (=limestone filler+slag).

**Figure 6:**
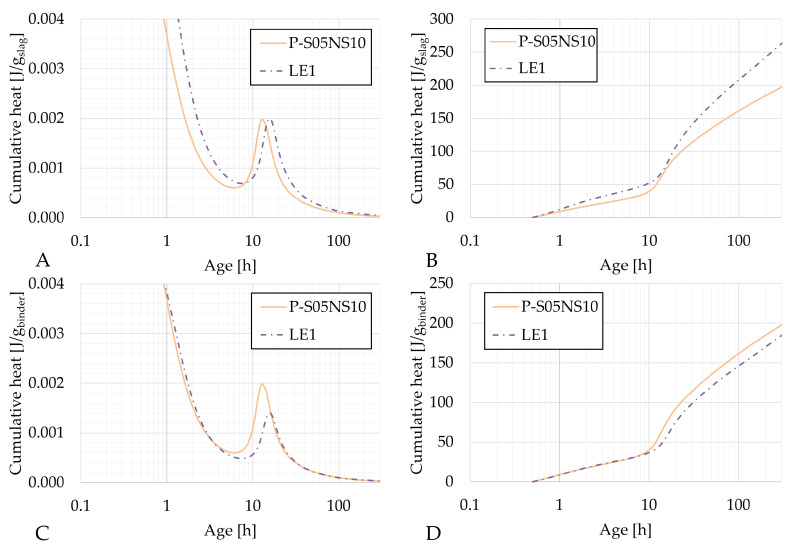
Results of the reaction kinetics followed through isothermal calorimetry for P-S05NS10 and LE1: (**A**) Heat flow and (**B**) cumulative heat presented per gram of slag; (**C**) Heat flow and (**D**) cumulative heat presented per gram of binder (=limestone filler+slag).

Figure 4, Figure 5 and Figure 6 present the heat flow and cumulative heat results of each reference composition and their corresponding limestone filler substitution compositions: P-S05M2, LA2 and LA4; P-S05M8, LB2 and LB4; and P-S05NS10 and LE1, respectively. The results are presented per gram of slag in figures A and B and per gram of binder (slag + limestone filler) in figures C and D. In the literature, limestone filler is often considered as an inert material [46,47]. Therefore, when expressed per gram of slag, the results should theoretically remain unchanged. However, since part of the slag is replaced with limestone filler, the solution-to-slag ratio effectively increases with higher substitution rates, leading to a slight increase in heat production [6]. As a result, the impact of the substitution rate cannot be accurately assessed using this approach. To improve the analysis, the results are also presented per gram of binder [22].

The peak observed in the results corresponds to the second peak typically seen in similar studies. This peak represents the formation of reaction products, such as calcium aluminosilicate hydrates [30,37,48]. The first peak, also called the dissolution peak, is not correctly monitored due to the ex situ mixing and has therefore been removed. This peak is related to the dissolution of the reactants, particularly the breakdown of bonds in the slag, the wetting of the particles, and the formation and interaction of units of silicate with Ca^2+^ and Na^+^ ions [37,38,39].

In each case, the second peak of the heat flow occurs slightly later for the limestone filler compositions compared to the reference compositions.

The heat flow per gram of binder for P-S05M2 decreases slightly with increasing limestone substitution, indicating that the limestone filler is either inert, or reacts less than slag. In addition, the increase in heat flow per gram of slag with higher substitution rates can be attributed to the effectively higher solution-to-slag ratio when more slag is replaced. Lacante et al. [6] found that an increase in the solution-to-slag ratio slightly increased the heat flow, with a more significant impact on the cumulative heat. For a 15% substitution, the solution-to-slag ratio becomes 0.58, while a 30% substitution results in a solution-to-slag ratio of 0.71. These values remain lower than the S/B = 0.8 studied by Lacante et al. Moreover, the addition of limestone filler to AAM leads to increased reaction and extended chain length of the reaction products [12]. It was also shown that limestone filler substitution resulted in decreased amounts of unreacted binder while the hydration product ratio was increased. This suggests that slag may exhibit increased reactivity in the presence of limestone filler [22,49].

In both ways of presenting the results for the P-S05M8 case, the second peak is lower when limestone filler is added. However, the cumulative heat per gram of slag increases with higher substitution rates, which can be attributed to the increase in the solution-to-slag ratio, as mentioned earlier. It is important to note that, in this case, the heat flow is still slightly lower than that of the reference P-S05M8. This suggests that replacing slag with a semi-inert material might influence the chemical reaction, especially when the reaction is fast.

For the P-S05NS10 composition, replacing slag with limestone filler does not appear to affect the heat flow per gram of slag. Instead, it primarily delays the reaction, leading to a broader peak over time and ultimately resulting in higher cumulative heat. Interestingly, the cumulative heat per gram of binder remains superimposed during the first ten hours before separating. At that point, the substitution further delays the reaction’s progression. However, once the cumulative heat increase resumes, it increases at the same rate as in the reference composition.

The ultimate heat was estimated based on the cumulative heat curves. Two methods were utilized [29,50,51]: polynomial fitting to the inverse of the square root of the age and exponential fitting using the Freiesleben Hansen and Pedersen model.

The initial approach involves plotting cumulative heat as a function of the inverse square root of age. A second-degree polynomial equation is then fitted within a specified age range, determined based on the $R^{2}$ (see Equation (3)). For each composition, this fitting interval is set between 25 h and 314 h. The resulting fitting parameters *a*, *b* and $Q_{\infty ,1}$, along with their associated error, are presented in Table A1.

(3) $$Q\left(t^{-\frac{1}{2}}\right)=a\cdot {\left(t^{-\frac{1}{2}}\right)}^{2}-b\cdot t^{-\frac{1}{2}}+Q_{\infty ,1}$$

The other method is based on an adaptation of the model of Freiesleben Hansen and Pedersen due to the multi-curvature nature of the data [52,53,54], see Equation (4).

(4) $$Q\left(t\right)=Q_{1}\cdot exp\left(-{\left(\frac{\tau_{1}}{t}\right)}^{a_{1}}\right)+Q_{2}\cdot exp\left(-{\left(\frac{\tau_{2}}{t}\right)}^{a_{2}}\right)$$

In this equation, *a* as well as τ are parameters depending on the material that rule the curve’s curvature and the intercept of the curve, respectively; and $Q_{1}+Q_{2}=Q_{\infty },2$.

The fitting process was performed by minimizing the sum of squares. The equation was applied to the age interval [0.5 h, 314 h]. The ultimate heat was computed both per gram of slag and per gram of binder; these values can be found in Table 6. Table A1 presents the values of all the fitting parameters along with the associated error.

The two methods yielded relatively different results. The increase in limestone filler substitution resulted in a higher maximum heat per gram of slag (which is in line with findings in Zhang et al. [55]), whereas the opposite trend was observed for the ultimate heat per gram of binder. However, the ultimate heat was consistently higher for LE1 compared to P-S05NS10, except for $Q_{\infty ,2,slag}$. For these compositions, the exponential method resulted in notably high values, suggesting that it may not be the most suitable approach for materials activated with sodium silicate. This is likely due to the disproportionate multi-curvature behavior observed in the cumulative heat curves, which is not the case for compositions with NaOH.

The ultimate heat can be used to compute the degree of reaction by Equation (5). This allows comparision of autogenous strain and the coefficient of thermal expansion as a function of the degree of reaction.

(5) $$DOR\left(t\right)=\frac{Q(t)}{Q_{\infty }}$$

#### 3.2.2. Autogenous Strains

**Figure 7:**
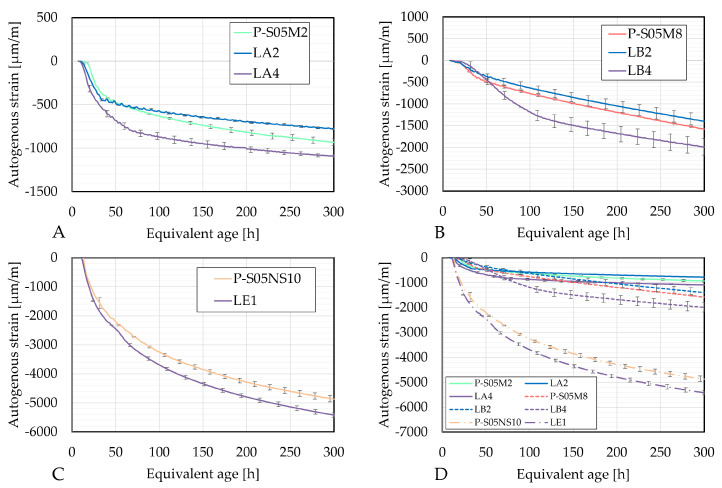
Autogenous strains as a function of the age: (**A**) P-S05M2 reference composition, (**B**) P-S05M8 reference composition, (**C**) P-S05NS10 reference composition, (**D**) Summary of all curves.

Figure 7 compares the autogenous strain of each composition relative to the reference compositions, with respect to the age. The autogenous strain curves were initialized based on the knee-point method. Time zero corresponds to the age at which the rate of autogenous strain reaches zero [56] or when it reaches its maximum in the case it does not reach zero [6]. This point corresponds to the solid-to-fluid state transition. Each initialization age as well as the corresponding DOR (calculated with the ultimate heats obtained in Table 6) is shown in Table 7.

All compositions undergo shrinkage. This behavior is primarily influenced by several factors. First, these materials possess a dense pore structure leading to high surface tension. Consequently, the higher degree of saturation results in an increased capillary pressure [43]. Additionally, alkali-activated slag materials exhibit a higher deformability because of the highly viscous nature of the formed C-A-S-H gels [57]. Furthermore, polycondensation between gel units occuring during the formation of the solid network reduces the distance between solid particles [58]. Lastly, shrinkage is further influenced by force imbalances. As the reaction progresses, the repulsive steric-hydration forces decrease. This is related to the decrease in the ion concentration in the pore solution. This happens while the attractive forces between the particles of the gel do not change [59].

A 15% limestone filler substitution reduces the autogenous shrinkage. It might be related to the increased solution-to-slag ratio, which reduces the self-desiccation shrinkage because of reduced capillary pressure and changes in the pore size distribution [8]. A 30% limestone filler substitution, however, increases the autogenous shrinkage, possibly due to the nucleation effect. The finer grains of limestone filler, compared to slag, fill voids while providing an additional surface area for the nucleation during the reaction [22,24]. Additionally, higher amounts of C-A-S-H gels have been reported when increasing the limestone filler substitution rate from 0 % to 10 % [21] or even up to 30 % [12]. This results in a higher deformability of the material [57].

Interestingly, the rate of shrinkage after 100 h is reduced when a higher limestone filler substitution rate is used for the NaOH-activated compositions. This might be related to the dilution effect or the delayed reaction process [21].

The addition of limestone filler to the mix does not result in swelling as observed for PC pastes [14].

**Figure 8:**
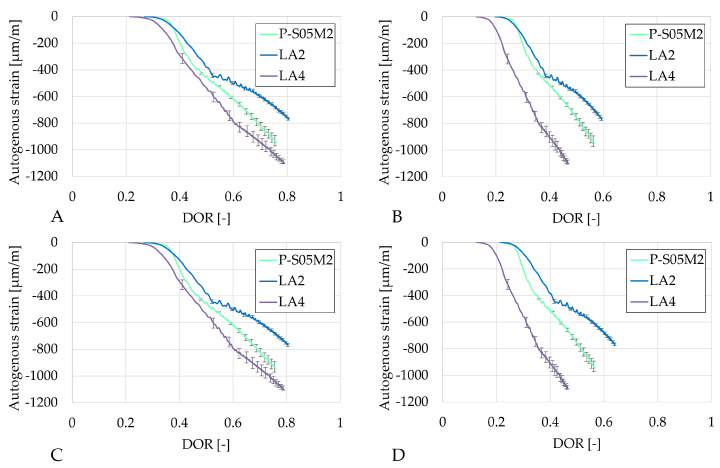
Results of the autogenous strain of the S05M2 reference presented as function of the DOR: (**A**) computed with Q_*∞*,1,slag_, (**B**) computed with Q_*∞*,2,slag_, (**C**) computed with Q_*∞*,1,binder_, (**D**) computed with Q_*∞*,2,binder_.

Figure 8 presents the autogenous strain as a function of the degree of reaction computed using the four ultimate heat values obtained in Table 6. The results based on the same methods are very similar. In each case, LA4 evolves the fastest with respect to the degree of reaction. Both compositions with limestone filler exhibit a change in rate (around DOR = 0.5 for LA2 and DOR = 0.6 for LA4, in Figure 8A), whereas the reference composition does not show such a distinct transition. After this point, the rate of autogenous shrinkage is reduced with respect to the rate in the reference composition.

**Figure 9:**
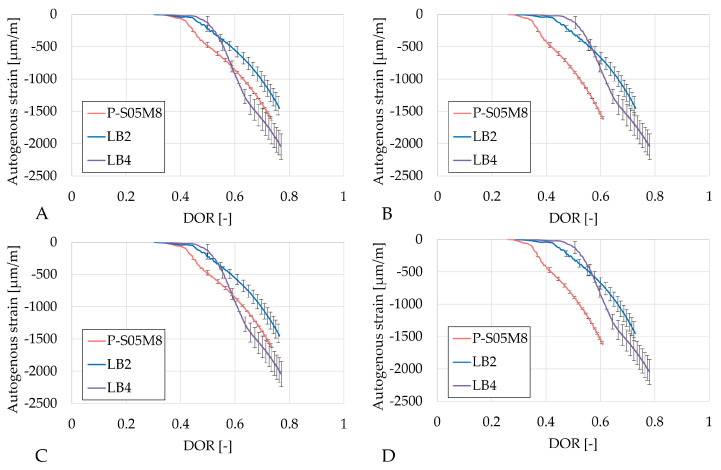
Results of the autogenous strain of the S05M8 reference presented as a function of the DOR: (**A**) computed with Q_*∞*,1,slag_, (**B**) computed with Q_*∞*,2,slag_, (**C**) computed with Q_*∞*,1,binder_, (**D**) computed with Q_*∞*,2,binder_.

Figure 9 presents the autogenous strain as a function of the degree of reaction computed with the four ultimate heat values obtained in Table 6. Similarly to the S05M2 compositions, results based on the same methods show comparable results. As the rate of autogenous strain decreases over the degree of reaction for LB2 and LB4, the rate of the autogenous shrinkage of the reference compositions P-S05M8, in contrast, increases. This suggests that a higher substitution rate reduces later age shrinkage, which still constitutes a significant portion of the overall shrinkage in such materials [60].

**Figure 10:**
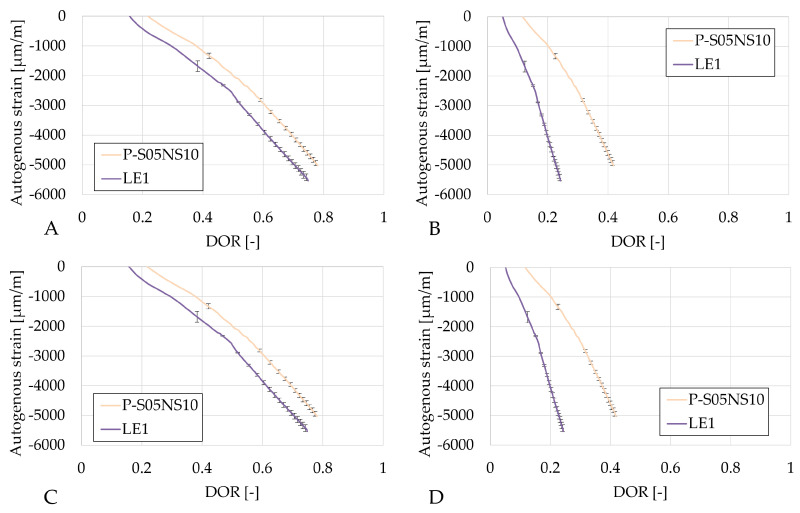
Results of the autogenous strain of the S05NS10 reference presented as a function of the DOR: (**A**) computed with Q_*∞*,1,slag_, (**B**) computed with Q_*∞*,2,slag_, (**C**) computed with Q_*∞*,1,binder_, (**D**) computed with Q_*∞*,2,binder_.

The results in Figure 10 reveal that an increase in limestone filler substitution in P-S05NS10 increases the autogenous shrinkage.

#### 3.2.3. Evolution of the Coefficient of Thermal Expansion

**Figure 11:**
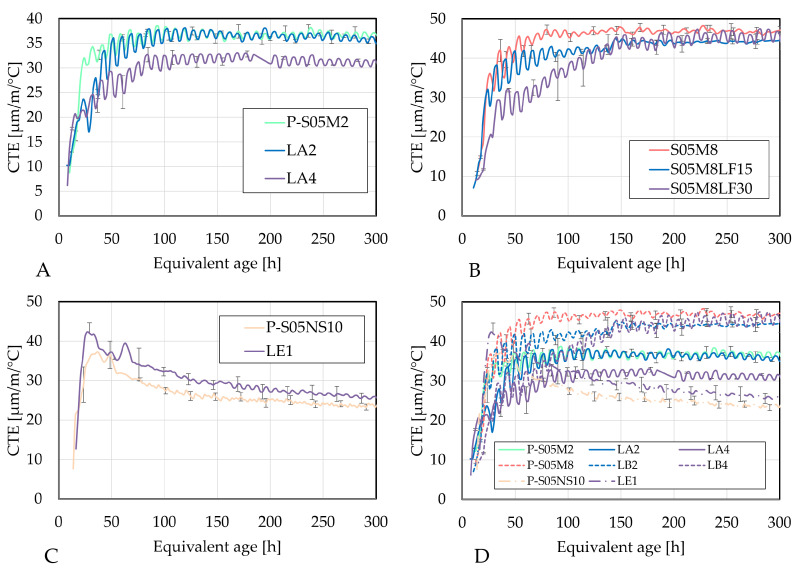
Evolution of the coefficient of thermal expansion with respect to age: (**A**) P-S05M2 as reference (Cat. A), (**B**) P-S05M8 as reference (Cat. B), (**C**) P-S05NS10 as reference (Cat. E), (**D**) Curves summary.

Due to the high coefficient of thermal expansion of alkali-activated materials [6], it is important to investigate the potential mitigating effect of limestone filler. Figure 11 presents the CTE as a function of age for each composition.

When 2 M NaOH is used, substituting 15% of slag with limestone filler primarily delays the CTE development, with a slight decrease observed at 300 h. However, increasing the substitution to 30% not only delays the development but also reduces the CTE by approximately 5 µm/m/°C. The delay might be related to the increase in the solution-to-slag ratio which delays the development of the CTE [29].

For compositions activated with 8 M NaOH, substituting 15% of slag with limestone filler reduces the CTE development, while a 30% substitution primarily delays it without significantly decreasing the final CTE value, which can be attributed to the delay in reaction observed in the heat flow results.

The reduction in the CTE with limestone filler substitution is attributed to its filler effect. The addition of limestone filler reduces porosity, leading to a lower CTE. Furthermore, the CTE of limestone filler (8 µm/m/°C) is lower compared to that of cement and slag [8].

**Figure 12:**
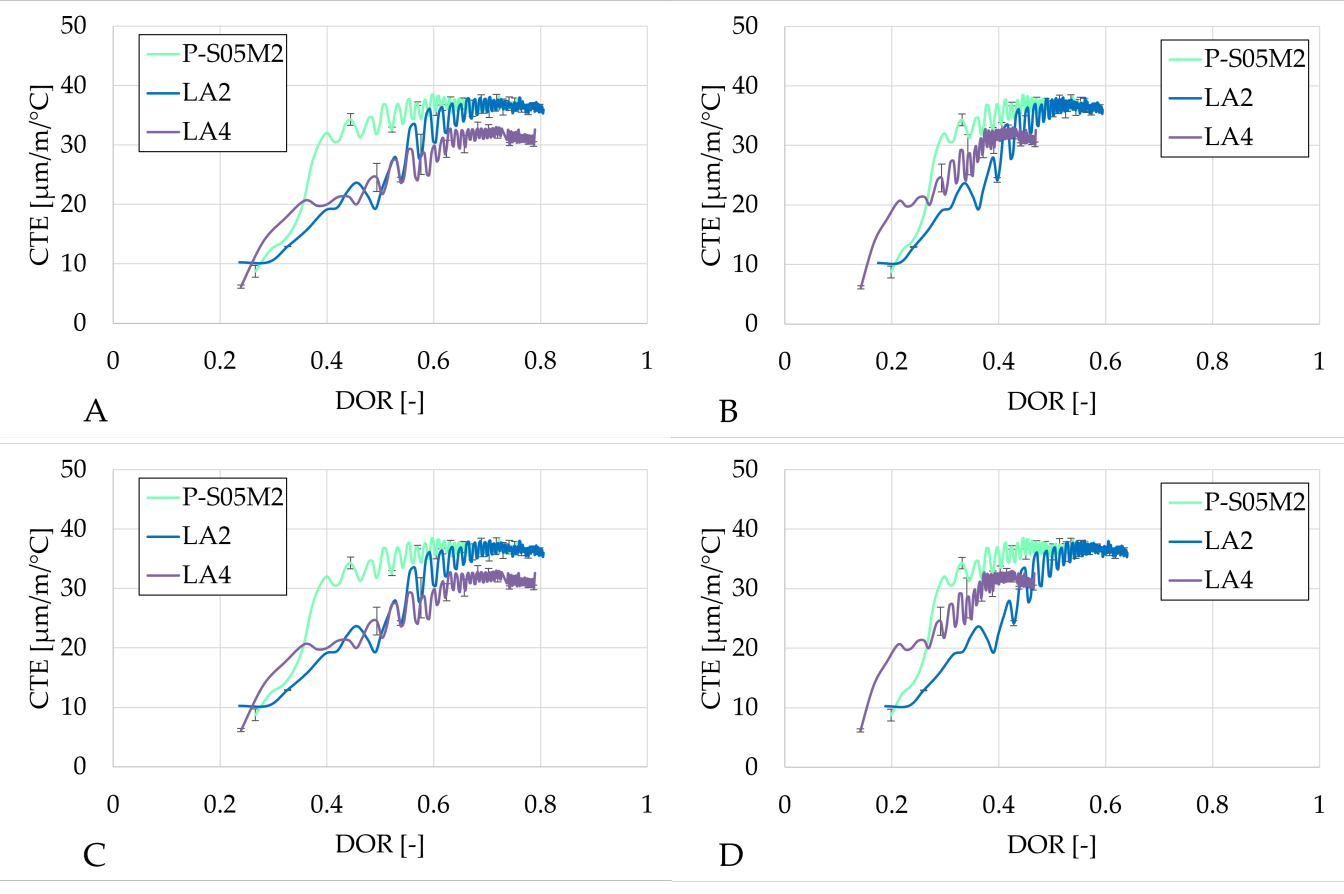
Results of the the coefficient of thermal expansion of the S05M2 reference presented as a function of the DOR: (**A**) computed with Q_*∞*,1,slag_, (**B**) computed with Q_*∞*,2,slag_, (**C**) computed with Q_*∞*,1,binder_, (**D**) computed with Q_*∞*,2,binder_.

**Figure 13:**
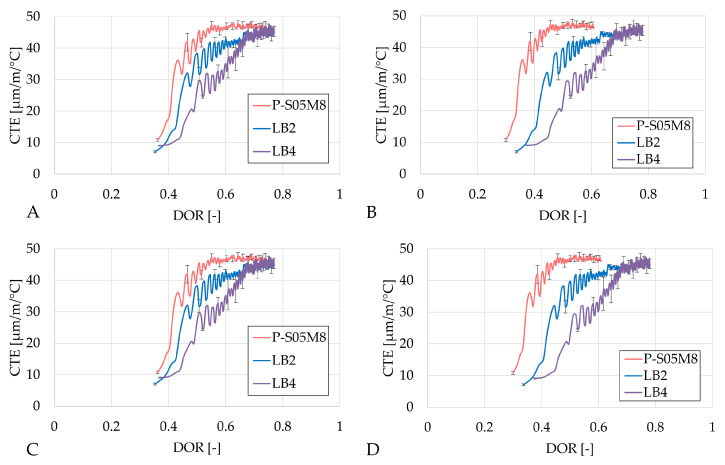
Results of the the coefficient of thermal expansion of the S05M8 reference presented as a function of the DOR: (**A**) computed with Q_*∞*,1,slag_, (**B**) computed with Q_*∞*,2,slag_, (**C**) computed with Q_*∞*,1,binder_, (**D**) computed with Q_*∞*,2,binder_.

**Figure 14:**
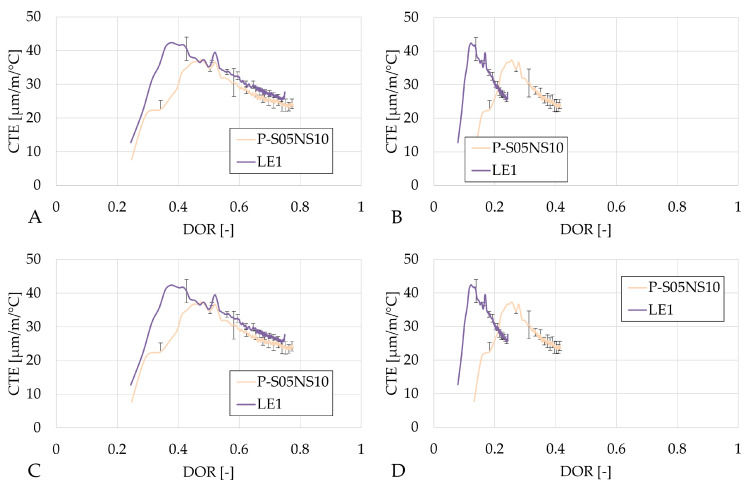
Results of the the coefficient of thermal expansion of the S05NS10 reference presented as a function of the DOR: (**A**) computed with Q_*∞*,1,slag_, (**B**) computed with Q_*∞*,2,slag_, (**C**) computed with Q_*∞*,1,binder_, (**D**) computed with Q_*∞*,2,binder_.

In terms of the degree of reaction, the CTE evolves in a very similar manner for the same method of determination of the ultimate heat values, just as for the autogenous strain results (see Figure 12, Figure 13 and Figure 14).

In the case of activating with NaOH, increasing the substitution rate delays the evolution of the CTE with respect to the degree of reaction, which is beneficial for the materials because of the strength development. This delay reduces the risk of cracking due to thermal strains, making the material less prone to damage.

When the sodium silicate activator is used, both the evolution rate and the overall CTE values increase.

## 4. Conclusions and Perspectives

1. The initial study considered the effect of different rates of replacement on the workability as well as on the compressive strength of the material. Five of the 24 initially proposed compositions were investigated in greater detail. At 7 days of age, LA2 and LB2 showed a reduction smaller than 13% for their compressive strength with a substitution of 15%, while LA4, LB4, and LE1 showed a decrease of less than 23% for a 30% substitution rate compared to their reference compositions. This resulted in compressive strengths at 7 days of 15.8 MPa, 30.8 MPa, 15.0 MPa, 28.4 MPa, and 81.8 MPa, respectively.
2. Isothermal calorimetry results indicated that limestone filler plays a role in the reaction. An increased substitution rate raised the solution-to-slag ratio (dilution effect), leading to a higher reaction peak; while also slowing down the reaction. In addition, slag might react more in the presence of limestone filler because of the nucleation effect (more nucleation surface available). The addition of limestone filler might result in the formation of more reaction products and extended length chains.
3. Autogenous shrinkage decreased with a 15% limestone filler substitution, likely due to the increased solution-to-slag ratio. This could reduce the self-desiccation shrinkage by decreasing capillary tension and delaying the reaction process. In contrast, higher autogenous shrinkage at increased substitution levels may be linked to the nucleation effect. In the end, the compositions containing limestone filler exhibited a reduced rate of autogenous shrinkage compared to the reference composition.
4. The substitution of limestone filler primarily delayed the development of the coefficient of thermal expansion, resulting in slightly lower results at 300 h. Notably, LA4 exhibited a CTE that was 5 µm/m/°C lower than the reference.

Future research will investigate the substitution by metakaolin in similar compositions. Perspectives include the investigation of the microstructure to help and explain the autogenous strains results.

## Figures and Tables

**Table 1:** Chemical composition [%] of the blast-furnace slag and limestone filler in mass percent. LOI = loss on ignition.

Material	SiO_2_	Al_2_O_3_	Fe_2_O_3_	CaO	K_2_O	MgO	TiO_2_	SO_3_	Na_2_O	BaO	MnO	SrO	P_2_O_5_	LOI
BFS	34.20	12.86	0.35	39.95	0.62	7.91	1.13	1.88	0.50	0.13	0.30	/	/	/
LF	0.31	0.11	0.09	55.03	0.02	0.42	0.01	0.09	0.09	/	/	0.03	0.01	43.78

**Table 2:** Compositions used as reference, where P-SYYMX is a paste with S/B = Y.Y and its solution has a concentration = X mol/L.

Ref Compositions	Alkaline Solution	S/B Ratio [-]	Concentration [mol/L]	Water/Alkaline Solution [-]
P-S05M2	NaOH	0.5	2	0
P-S05M8				
P-S08M2	NaOH	0.8	8	0
P-S08M8				
P-S05NS10	Na_2_SiO_3_	0.5	10	1/1
P-S08NS10		0.8		1/1

**Table 3:** Compositions with limestone filler. LFXXSYYMZ is a paste with S/B = Y.Y, the solution has a concentration = Z mol/L and the slag is substituted by XX %.

ID	Compositions	Alkaline Solution	Limestone Filler Ratio [%]	S/B Ratio [-]	Concentration [mol/L]	Water/Alkaline Solution [-]
LA1	LF10S05M2	NaOH	10	0.5	2	0
LA2	LF15S05M2		15			
LA3	LF20S05M2		20			
LA4	LF30S05M2		30			
LA5	LF40S05M2		40			
LA6	LF50S05M2		50			
LB1	LF10S05M8	NaOH	10	0.5	8	0
LB2	LF15S05M8		15			
LB3	LF20S05M8		20			
LB4	LF30S05M8		30			
LB5	LF40S05M8		40			
LB6	LF50S05M8		50			
LC1	LF10S08M2	NaOH	10	0.8	2	0
LC2	LF20S08M2		20			
LC3	LF30S08M2		30			
LC4	LF40S08M2		40			
LC5	LF50S08M2		50			
LD1	LF10S08M8	NaOH	10	0.8	8	0
LD2	LF20S08M8		20			
LD3	LF30S08M8		30			
LD4	LF40S08M8		40			
LD5	LF50S08M8		50			
LE1	LF30S05NS10	Na_2_SiO_3_	30	0.5	10	1/1
LE2	LF50S08NS10		50	0.8		

**Table 4:** Reduction in the compressive strength [%] relative to the reference composition, depending on the rate of substitution, at 7 days of age.

LF ratio	P-S05M2	P-S05M8	P-S08M2	P-S08M8	P-S05NS10	P-S08NS10
10%	1.88	5.42	−10.29	−12.02	/	/
15%	10.06	12.37	/	/	/	/
20%	26.95	9.02	−3.88	−2.17	/	/
30%	15.05	19.25	22.50	−11.93	22.64	/
40%	42.58	30.29	32.45	12.20	/	/
50%	48.86	52.23	51.78	41.30	/	51.20

**Table 5:** Boost in the compressive strength [%] relative to the reference composition, determined with Equation (2), depending of the rate of substitution, at 7 days.

LF Ratio	P-S05M2	P-S05M8	P-S08M2	P-S08M8	P-S05NS10	P-S08NS10
10%	9.02	5.09	22.54	24.47	/	/
15%	5.81	3.09	/	/	/	/
20%	−8.69	13.73	29.85	27.72	/	/
30%	21.36	15.35	10.72	59.91	10.52	/
40%	−4.30	16.18	12.58	46.33	/	/
50%	2.27	−4.47	−3.55	17.39	/	−2.40

**Table 6:** Estimations of the ultimate heat [J/g].

		P-S05M2	LA2	LA4	P-S05M8	LB2	LB4	P-S05NS10	LE1
Per gram of slag	Q_*∞*,1,slag_	220.20	242.01	288.86	345.99	341.20	396.53	256.80	354.63
	Q_*∞*,2,slag_	295.33	328.02	486.07	417.61	358.01	391.32	479.62	1096.86
Per gram of binder	Q_*∞*,1,binder_	220.20	205.71	202.20	345.99	290.20	277.57	256.80	248.24
	Q_*∞*,2,binder_	295.33	258.64	342.52	417.61	304.31	273.92	479.62	766.20

**Table 7:** Age of initialization $t_{0}$ for autogenous strain and corresponding DOR.

Composition	$t_{0}$ [h]	DOR_*∞*,1,slag_ [-]	DOR_*∞*,2,slag_ [-]	DOR_*∞*,1,binder_ [-]	DOR_*∞*,2,binder_ [-]
P-S05M2	11.47	0.287	0.214	0.287	0.214
LA2	8.80	0.267	0.197	0.268	0.213
LA4	6.98	0.212	0.126	0.212	0.125
P-S05M8	9.89	0.314	0.260	0.314	0.260
LB2	7.97	0.303	0.288	0.302	0.288
LB4	9.90	0.325	0.330	0.325	0.330
P-S05NS10	12.75	0.217	0.116	0.217	0.116
LE1	11.06	0.157	0.050	0.157	0.051

## Data Availability

The original contributions presented in this study are included in the article. Further inquiries can be directed to the corresponding author.

## Abbreviations

AAM	Alkali-activated materials
CTE	Coefficient of thermal expansion
DOR	Degree of reaction
LF	Limestone filler
PC	Portland cement
S/B	Solution-to-binder mass ratio

## Appendix A

**Table A1:** Fitting results for the ultimate heat estimations.

			P-S05M2	LA2	LA4	P-S05M8	LB2	LB4	P-S05NS10	LE1
Per gram of slag	Q_*∞*,1,slag_	a	2256.20	1290.20	2200.60	5103.60	4204.70	4632.00	2084.10	3601.20
		b	−1089.30	−893.09	−1219.90	−1968.80	−1709.10	−1934.40	−1160.90	−1820.80
		c = Q_*∞*,1,slag_	220.20	242.01	288.86	345.99	341.20	396.53	256.80	354.63
		R^2^	0.9984	0.9992	0.9986	0.9954	0.9967	0.9969	0.9992	0.9978
Per gram of slag	Q_*∞*,2,slag_	Q1	63.68	142.34	158.21	217.51	226.74	278.77	59.05	70.55
		τ _1_	5.26	8.42	7.86	5.65	5.73	6.87	15.33	18.58
		a_1_	1.03	0.81	0.85	0.64	0.72	0.71	2.45	2.53
		Q_2_	231.65	185.67	327.86	200.10	131.27	112.55	420.58	1026.31
		τ _2_	156.89	442.07	1388.60	504.91	338.21	295.43	472.37	6548.13
		a_2_	0.32	0.28	0.25	0.60	0.72	0.86	0.22	0.17
		error	31.82	45.61	31.82	50.06	51.76	104.84	426.88	608.90
		Q_*∞*,2,slag_	295.33	328.02	486.07	417.61	358.01	391.32	479.62	1096.86
Per gram of binder	Q_*∞*,2,slag_	a	2256.20	1096.70	1540.40	5103.60	3574.00	3242.40	2084.10	2520.80
		b	−1089.30	−759.12	−853.94	−1968.80	−1452.70	−1354.10	−1160.90	−1274.60
		c = Q_*∞*,1,slag_	220.20	205.71	202.20	345.99	290.20	277.57	256.80	248.24
		R^2^	0.9984	0.9992	0.9986	0.9954	0.9967	0.9969	0.9992	0.9978
Per gram of binder	Q_*∞*,2,slag_	Q1	63.68	110.91	111.16	217.51	192.73	195.14	59.05	49.33
		τ _1_	5.26	8.06	7.88	5.65	5.73	6.87	15.33	18.58
		a_1_	1.03	0.84	0.84	0.64	0.72	0.71	2.45	2.54
		Q_2_	231.65	147.72	231.36	200.10	111.58	78.79	420.58	716.87
		τ _2_	156.89	208.06	1455.55	504.91	338.21	295.43	472.37	6463.27
		a_2_	0.32	0.31	0.25	0.60	0.72	0.86	0.22	0.17
		error	31.82	32.74	15.59	50.06	37.40	51.37	426.88	298.38
		Q_*∞*,2,slag_	295.33	258.64	342.52	417.61	304.31	273.92	479.62	766.20
